# S1‐Leitlinie Infizierte Zehenzwischenraum‐Intertrigo (sogenannter „gramnegativer Fußinfekt“)

**DOI:** 10.1111/ddg.70040

**Published:** 2026-06-04

**Authors:** Christoph Zeyen, Dietrich Abeck, Karsten Becker, Joachim Dissemond, Birgit Kahle, Hans Peter Lorenzen, Bettina Löffler, Andreas Maier‐Hasselmann, Till Mittank‐Weidner, Alexander Nast, Cord Sunderkötter

**Affiliations:** ^1^ Klinik für Dermatologie Venerologie und Allergologie Division of Evidence Based Medicine in Dermatology (dEBM) Charité – Universitätsmedizin Berlin corporate member of Freie Universität Berlin and Humboldt‐Universität zu Berlin Berlin Deutschland; ^2^ Hautzentrum Nymphenburg München Deutschland; ^3^ Friedrich Loeffler‐Institut für Medizinische Mikrobiologie Universitätsmedizin Greifswald Greifswald Deutschland; ^4^ Klinik und Poliklinik für Dermatologie Venerologie und Allergologie Universitätsklinikum Essen Essen Deutschland; ^5^ Klinik für Dermatologie Venerologie und Allergologie Universitäts‐Klinikum Schleswig‐Holstein (UKSH) Campus Lübeck Lübeck Deutschland; ^6^ Klinik für Nephrologie Angiologie und Rheumatologie Klinikum Siloah Klinikum Region Hannover Hannover Deutschland; ^7^ Institut für Medizinische Mikrobiologie Universitätsklinikum Jena Jena Deutschland; ^8^ Klinik für Gefäßchirurgie München Klinik Bogenhausen München Deutschland; ^9^ Klinik und Poliklinik für Dermatologie Venerologie und Allergologie Universitätsklinikum Leipzig Leipzig Deutschland; ^10^ Universitätsklinik und Poliklinik für Dermatologie und Venerologie Universitätsmedizin Halle Martin‐Luther‐Universität Halle‐Wittenberg Halle (Saale) Deutschland; ^11^ MSB Medical School Berlin Hochschule für Gesundheit und Medizin Berlin Deutschland

**Keywords:** Definition, Empfehlungen, Gramnegativer Fußinfekt, infizierte Zehenzwischenraum‐Intertrigo, Therapie, Definition, gram‐negative foot infection, infected interdigital intertrigo, recommendations, therapy

## Abstract

Die infizierte Zehenzwischenraum‐Intertrigo bezeichnet eine exsudative, mazerierende oberflächliche Mischinfektion der Zehenzwischenräume, bei der gramnegative Bakterien (vor allem *Pseudomonas aeruginosa*, *Enterobacterales*) hervorstechen, aber regelmäßig auch grampositive Erreger (*Staphylococcus [S.] aureus*, koagulasenegative Staphylokokken, Streptokokken, Enterokokken) und Pilze (Dermatophyten, Hefen) zu finden sind. Der auch international gebrauchte Begriff ersetzt die bisher im Deutschen verwendete Bezeichnung „gramnegativer Fußinfekt“, da letztere missverständlich den Eindruck erweckt, auch das Weichgewebe des Fußes sei mit gramnegativen Bakterien infiziert. Aus einer oberflächlich infizierten Zehenzwischenraum‐Intertrigo kann zwar eine unkomplizierte oder komplizierte Phlegmone hervorgehen, doch deren primärer Erreger sind bei Immunkompetenten meist grampositive Bakterien, insbesondere *S. aureus*. Komplizierte Verläufe sowie die Entstehung der Zehenzwischenraum‐Intertrigo selbst werden durch Diabetes mellitus, Polyneuropathie und Gefäßerkrankungen begünstigt. Die Diagnose stützt sich klinisch auf mazerierte, rot‐weißliche Erosionen, manchmal Ulzera, mit gelb‐grünlichem Exsudat, putridem Geruch, Ödem und häufig Schmerzen. Oberflächliche Hautabstriche sind diagnostisch nicht immer erforderlich oder aussagekräftig. Therapeutisch steht eine strukturierte Lokaltherapie (antientzündlich, antiseptisch, antimykotisch, ödemreduzierend, austrocknend) im Vordergrund. Systemische Antibiotikatherapie ist nur bei Weichgewebeinfektionen indiziert, die initial oft unkomplizierte Phlegmone darstellen und dann entsprechend mit Antibiotika gegen *S. aureus* behandelt werden. Aufgrund der häufigen Begleitkrankheiten sind jedoch frühzeitige vaskuläre Abklärung, Bildgebung bei Verdacht auf Infektionsausbreitung sowie chirurgische Evaluation bei tiefer Infektion oder Nekrose wichtig.

## EINLEITUNG UND BEGRIFFSDEFINITION

Der Begriff *gramnegativer Fußinfekt* wird traditionell in der deutschen klinischen Fachsprache genutzt, ist aber weder grammatikalisch korrekt noch inhaltlich präzise.

In der Erregerdiagnostik werden bei dieser Entität meist unterschiedliche Mikroorganismen nachgewiesen, wobei es auf alleiniger Basis dieser Befunde unklar bleibt, welcher Erreger ursächlich und/oder infektionsunterhaltend ist. Aus den klinisch relevanten Hautveränderungen werden neben gramnegativen Bakterien oft auch grampositive Bakterien und Pilze isoliert (siehe Kapitel „Erregerspektrum“).^1^ Für eine daraus hervorgehende Infektion der Weichgewebe sind bei immunkompetenten Patienten in der Regel nicht die aus der Intertrigo isolierten gramnegativen Erreger, sondern typischerweise *Staphylococcus (S.) aureus* verantwortlich, sodass Staphylokokken‐wirksame Antibiotika zur Behandlung ausreichen.^1^, ^2^ Daher ist der Begriff „gramnegativ“ irreführend. Zudem suggeriert das Wort „Infekt“ eine kurzzeitige selbstlimitierende Infektion und „Fußinfekt“ eine Infektion des gesamten Fußes. Es liegt aber eine lokal begrenzte Infektion ausgehend von Erosionen oder Ulzera vor, die in den Zehenzwischenräumen beginnt und dann oft in Form flächiger Erosionen beziehungsweise Ulzera auf den Fußrücken übergreifen kann. Die klinische Abgrenzung beispielsweise zum Erysipel oder zur Phlegmone erscheint im bisherigen Gebrauch des Begriffs nicht klar.

Die Leitliniengruppe schlägt daher vor, den Terminus „gramnegativer Fußinfekt“ aufzugeben und zu ersetzen. Es wird vorgeschlagen, über die Pathogenese des Krankheitsbildes eine neue Bezeichnung herzuleiten, die in ähnlicher Form auch international gebräuchlich ist. In der Regel sind die Zehenzwischenräume und der Vorfuß infiziert. Es liegt ursächlich ein Barrieredefekt vor – oft in Form eines meist flächigen Ulkus, ausgehend von Erosionen in den Zehenzwischenräumen im Sinne einer Intertrigo. Es wird daher vorgeschlagen, den Begriff infizierte Intertrigo der Zehenzwischenräume, kurz infizierte Zehenzwischenraum‐Intertrigo (IZI) zu verwenden, da somit das klinische Bild eindeutiger und nicht therapeutisch irreführend beschrieben wird. Im angloamerikanischen Sprachraum ist der Begriff *toe web infection/intertrigo* ebenfalls bekannt, wenngleich auch hier gelegentlich noch das Attribut *Gram‐negative* hinzugefügt wird, zum Beispiel *Gram‐negative bacterial toe web infection* (GNTWI) oder *Gram‐negative bacterial toe web intertrigo*.^3^, ^4^, ^5^

**ABBILDUNG 1:**
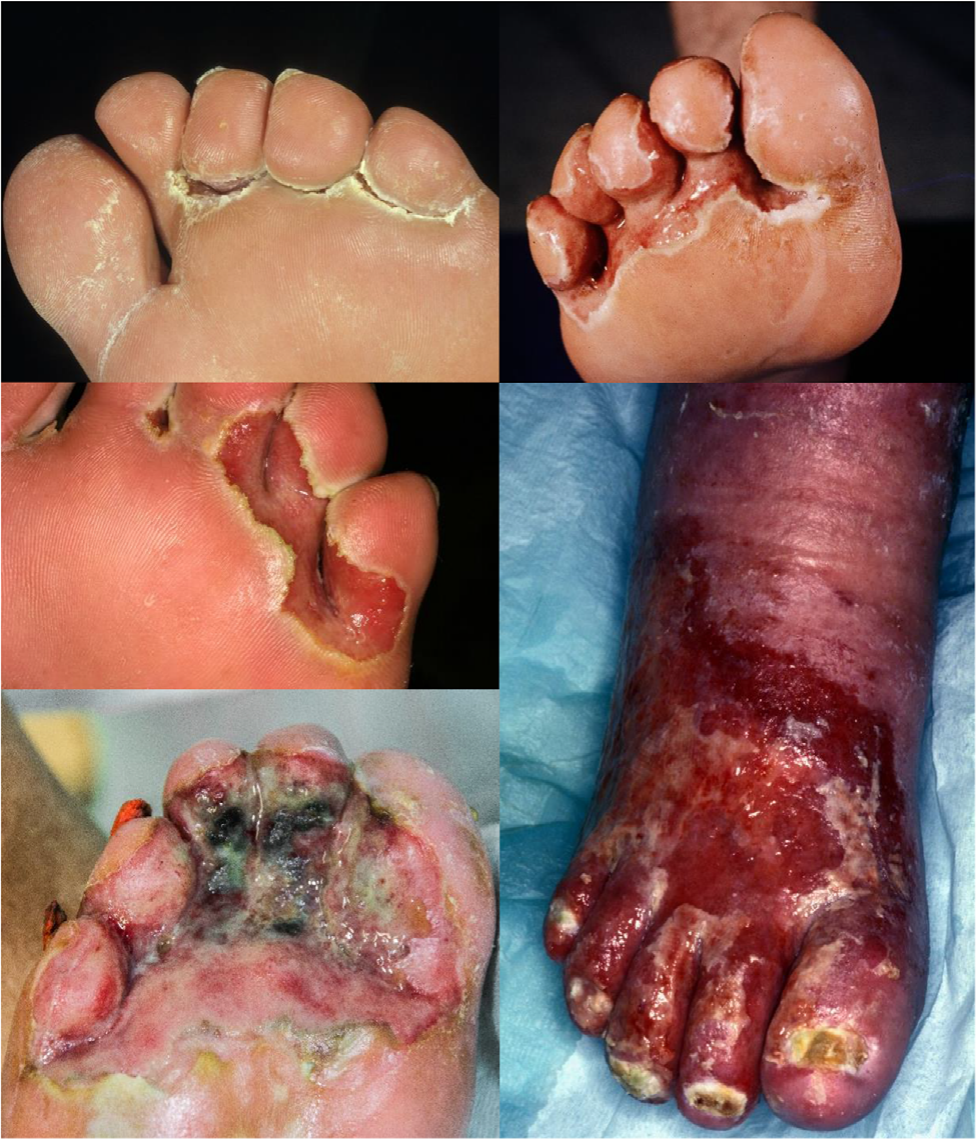
Klinische Fotos der infizierten Zehenzwischenraum‐Intertrigo (Wundzentrum der Klinik für Dermatologie, Universitätsklinikum Essen).

Klinisch typisch sind die vor allem durch gramnegative Besiedlung verursachte charakteristische Geruchsbildung (süßlich/putride) und die durch eine feuchte Mazeration bedingte Farbe (rot exsudativ, weißlich mazeriert) (Abbildung 1). Zusätzlich bestehen meist Ödeme und Schmerzen.

Oft liegen Begleitkrankheiten (Komorbidität) vor, wie Diabetes mellitus, Polyneuropathie oder periphere arterielle Verschlusskrankheit (pAVK), welche die lokale Infektion begünstigen.^6^ Als Ursache für den Barrieredefekt der Haut sind eine mazerierende interdigitale Fußmykose oder Traumata verantwortlich.

**Table:** 

**Definition: Infizierte Zehenzwischenraum‐Intertrigo (IZI) / sogenannter „gramnegativer Fußinfekt“**
Exsudative, infizierte Intertrigo der Zehenzwischenräume, die mit Mazerationen und Erosionen beginnt und im Verlauf ulzerierend auf den Vorfuß übergreifen kann. Üblicherweise geht das dermatologische Krankheitsbild mit dem kulturellen Nachweis unterschiedlicher grampositiver (Staphylokokken, Enterokokken, Streptokokken unter anderem) und gramnegativer (*Pseudomonas aeruginosa, Enterobacterales* unter anderem) Erreger sowie von Pilzen einher. Oftmals bestehen Begleitkrankheiten (Komorbidität), welche die Entstehung der IZI begünstigen. Die IZI kann in eine Weichgewebeinfektion (unkomplizierte oder komplizierte Phlegmone) übergehen, deren Verursacher bei immunkompetenten Patienten dann eher grampositive Bakterien wie *S. aureus* und nicht gramnegative Bakterien sind.

## ERREGERSPEKTRUM

Der Fuß ist besonderen Belastungen ausgesetzt und zeigt aus mehreren Gründen eine reichhaltige Besiedlung mit verschiedenen und zum Teil wechselnden Mikroorganismen. Die physiologische Besiedlung der Haut des Fußes mit Mikroorganismen (Mikrobiota) ist von einer hohen Komplexität. Es wird angenommen, dass es zu einer dynamischen Interaktion zwischen Mikrobiota und Wirt sowie innerhalb der Mikrobiota kommt.^7^

Faktoren, welche zu Unterschieden im Mikrobiom führen können, sind:

− eher trockene (zum Beispiel Fußrücken) und eher feuchte Regionen (zum Beispiel Zehenzwischenräume),
− unterschiedlich bakteriell kolonisierte Oberflächen, mit denen Füße Kontakt haben,
− Veränderungen des Mikroklimas durch Tragen von Strümpfen und unterschiedlich ventiliertem Schuhwerk sowie dadurch bedingte Unterschiede der lokalen Temperatur und Feuchtigkeit,^8^
− Art, Dauer und Lokalisation von akuten und chronischen Hauterkrankungen und Hautdefekten (inklusive oberflächlicher bis tiefer Wunden),
− Komorbiditäten des Wirtes einschließlich lokaler und/oder systemischer Immundefekte und Durchblutungsstörungen sowie antibiotisch/biozid wirkende Interventionen.^7^, ^9^ Am besten untersucht in diesem Zusammenhang sind die mikrobiellen Nachweise aus diabetischen Fußulzera (DFU).^9^, ^10^, ^11^

Die Ergebnisse von Mikrobiota‐Untersuchungen von Hautoberflächen sind stark von der Art und Qualität der Methodik (molekular und/oder kulturbasiert; optimal beide Verfahren) abhängig. Dazu gehören die Probengewinnung (Abstrich oder Gewebe) und die taxonomische Tiefe der Analysen.^10^, ^12^ Sie entsprechen nur bedingt den Erfahrungen, die auf der Untersuchung von Hautabstrichen oder anderen Untersuchungsmaterialien des Fußes im mikrobiologischen Routinelabor beruhen und die sich diagnostisch oft auf den Nachweis ätiopathogenetisch relevanter Mikroorganismen beschränkt haben. Untersuchungen gesunder Probanden beziehungsweise von Kontrollgruppen zeigen eine typische Besiedlung der Fußhaut mit Vertretern aus vier bakteriellen Reichen (Phyla), wie es generell für die Besiedlung der menschlichen Haut bekannt ist. Am häufigsten finden sich Bakterien der *Staphylococcaceae* und *Streptococcaceae* sowie *Pseudomonadota* (synonym *Proteobacteria*; unter anderem mit *Enterobacteriaceae*, *Moraxellaceae*, *Neisseriaceae* und *Pasteurellaceae*), *Actinomycetota* (synonym *Actinobacteria*; unter anderem mit *Corynebacteriaceae*, *Kytococcaceae, Micrococcaceae* und *Propionibacteriaceae*) und *Bacillota* (synonym *Firmicutes*; unter anderem mit *Bacillaceae*, *Clostridiaceae*, *Enterococcaceae*, und *Lactobacillaceae*), weniger häufig Arten der *Bacteroidota* (synonym *Bacteroidetes*).^9^, ^13^, ^14^, ^15^ Dominierend erscheinen am Fuß insgesamt koagulasenegative Staphylokokken‐Arten, auch in Kulturen aus den Zehenzwischenräumen (Nachweis in circa 75%), gefolgt von Corynebakterien (circa 45%) und Mikrokokken (circa 35%); gramnegative Arten finden sich bei etwa 10%.^15^, ^16^ Typische eukaryonte Vertreter auf der Fußhaut sind *Malassezia*‐Spezies gefolgt von *Candida*‐Arten aus dem Reich („*kingdom*“) der Pilze und *Demodex*‐Milben aus dem Reich der *Arthropoda*. Pilze sind zudem häufig an Hautinfektionen bei Menschen mit Diabetes beteiligt (Prävalenz von 84,6% bei Typ‐1‐Diabetes). Neben *Candida albicans* zählen hierzu auch Dermatophytosen der Zehenzwischenräume und der Nägel.^17^

Bei der infizierten Zehenzwischenraum‐Intertrigo (IZI) handelt es sich zumeist (22%–90%) um Mischinfektionen.^4^, ^18^, ^19^ Es wurden in kulturbasierten klinischen Studien aus den Erosionen oder Ulzera zumeist (22%–90%) mehrere verschiede Bakterien isoliert.^11^, ^12^, ^13^ Typischerweise fanden sich in diesen kulturbasierten klinischen Studien unter den grampositiven Mikroorganismen *S. aureus* und/oder koagulasenegative Staphylokokken (circa 8%–41%), *Streptococcus pyogenes* (circa 2%–5%) oder *Enterococcus faecalis* (circa 40%)^2^, ^18^ und unter den gramnegativen Bakterien am häufigsten Isolate von *Pseudomonas aeruginosa* (16%–86%), gefolgt von *Enterobacterales*‐Vertretern (insbesondere *Escherichia coli, Proteus mirabilis, Morganella morganii, Enterobacter cloacae, Klebsiella pneumoniae* und *Serratia marcescens*) und *Acinetobacter species* mit einer Häufigkeit von etwa 3%–40%.^5^, ^6^, ^18^, ^19^ In über 50% der Fälle können mikroskopisch Pilzstrukturen nachgewiesen werden, die – wenn kultivierbar – vorrangig als *Trichophyton (T.) rubrum* beziehungsweise *T. mentagrophytes* und als *Candida albicans* in Erscheinung treten.^3^, ^5^

In Ergebnissen von Mikrobiom‐Untersuchungen und kulturbasierten Analysen fanden sich somit häufig grampositive Bakterien wie *S. aureus* oder *Enterococcus faecalis* und nicht ausschließlich gramnegative Bakterien. Somit erscheint die Intention, die sich in dem Attribut „gramnegativer“ Fußinfekt ausdrückt, nicht gerechtfertigt.

## DIFFERENZIALDIAGNOSEN UND KOMPLIKATIONEN

Die Tabelle 1 ermöglicht einen Überblick über die relevanten Differenzialdiagnosen und mögliche Komplikationen. Die zentralen Unterschiede in der klinischen Präsentation sind aufgelistet. Es besteht kein Anspruch auf Vollständigkeit.

**TABELLE 1:** Differenzialdiagnosen und Komplikationen der infizierten Zehenzwischenraum‐Intertrigo.

**Ekthymata**
− scharf begrenzte, meist multiple, ulzerierende Infektionen der Haut durch *S. aureus* oder *S. pyogenes*, die meist an den Beinen und vereinzelt auf dem Fußrücken auftreten (häufig beidseitig) − bei Erwachsenen bestehen meist entweder akut begünstigende Bedingungen in Form von Okklusion bei feuchtwarmem Klima oder schlechten hygienischen Verhältnissen − initial follikulär gebundene Pustel, aus der sich eine gräulich‐gelbliche Kruste entwickelt − nach Lösen der Kruste flaches Ulkus mit scharf begrenzten Rändern und umgebendem Erythem − Ausbreitung binnen weniger Tage auf 1–3 cm, dann größenstabil
**Weichgewebeinfektionen des Fußes**
***Erysipel***
− Erreger sind meist β‐hämolysierende Streptokokken, am häufigsten der Gruppe A (*S. pyogenes*) und seltener der Gruppen B, C und G − ‐hellrotes, glänzendes, Erythem, scharf begrenzte Ränder, oft mit bogigen Ausläufern, keine Eiterbildung − es besteht eine Eintrittspforte, zum Beispiel Tinea pedis − erhöhtes CRP und Leukozytose − ‐Allgemeinsymptomatik (Frösteln, Schüttelfrost, Fieber), meist zu Beginn des Erythems − ‐ kann Folge einer infizierten Zehenzwischenraum‐Intertrigo (“gramnegativen Fußinfektion“) sein, aber deutlich seltener als eine Phlegmone
***Unkomplizierte Phlegmone***
− Erreger meist *S. aureus* − dunkleres, intensiver livides Erythem als beim Erysipel − Teigige Schwellung, unterschiedlich ausgeprägte Eiterbildung − Eintrittspforte (Ulkus) meist tiefer als bei Erysipel − nicht immer von Allgemeinsymptomatik begleitet (Schüttelfrost, Fieber) und wenn, dann nicht immer initial, sondern auch erst im Verlauf − oft zu Beginn auch keine deutlich erhöhten serologischen Werte zum Beispiel für CRP oder Leukozyten − kann Folge einer infizierten Zehenzwischenraum‐Intertrigo sein − ohne erschwerende Kofaktoren zum Beispiel pAVK, Diabetes mellitus mit Folgeschäden oder unzureichender Stoffwechselkontrolle (≠ komplizierte Phlegmone)
***Komplizierte oder schwere Phlegmone***
− Erreger meist *S. aureus* oder Infektion beziehungsweise Mischinfektionen mit anderen grampositiven Erregern sowie gramnegativen Erregern und Anaerobiern − bis zur Muskelfaszie oder zum Muskel reichende Weichgewebeinfektion − einhergehend mit schweren Grunderkrankungen oder anderen Faktoren, welche ein Ansprechen der Therapie erschweren (es gelten die Kriterien für die komplizierte Weichgewebeinfektion (*complicated soft tissue infection*) wie sie für klinische Studien von der FDA definiert wurden^20^) − zu den erschwerenden Grundkrankheiten oder Faktoren zählen: insuffizient eingestellter Diabetes mellitus, Bakteriämie, Neutropenie (Granulozytenzahl < 500/mm^3^), Leberzirrhose (Child‐Klassifikation B oder C), chronischer Alkoholabusus, Malnutrition oder immunsuppressive Therapie − kann unter obigen Risikofaktoren Folge einer infizierten Zehenzwischenraum‐Intertrigo sein
**Fußinfektionen bei Patienten mit meist insuffizient eingestelltem Diabetes mellitus (als eine Form der schweren, komplizierten Phlegmone)**: − Erregerspektrum enthält verhältnismäßig häufig gramnegative Bakterien und Anaerobier, zudem sind häufig bereits multiresistente Erreger ursächlich − Niereninsuffizienz und weitere die Immunantwort beeinträchtigende Komorbiditäten
− schnelle Ausbreitung in tiefe Gewebeschichten, angrenzende Sehnen und innerhalb der Scheiden, Gelenkkapseln, Knochen (Osteomyelitis) und in den Blutstrom aufgrund metabolischer, neuropathischer und angiopathischer Schäden infolge der chronisch erhöhten Blutzuckerspiegel: ○ verminderte Immunreaktion (unter anderem eingeschränkte Funktion der Neutrophilen) ○ Ischämie aufgrund arterieller Gefäßverschlüsse (Kapillarsystem bis hin zu großen peripheren Gefäßen) ○ chronische, wegen diabetischer Neuropathie erst spät erkannte DFU, an mechanisch belasteten Arealen wie zum Beispiel den Zehenzwischenräumen ○ hyperglykämisches, die Pathogenität von *S. aureus* förderndes Wundmilieu − kann unter obigen Risikofaktoren Folge einer infizierten Zehenzwischenraum‐Intertrigo sein
***Nekrotisierende Haut‐ und Weichgewebeinfektion Typ I***
− seltene aggressiv verlaufende Infektionen, in deren Verlauf es aufgrund der Pathogenität und Virulenzfaktoren (insbesondere Toxine und Enzyme) bestimmter Bakterien(‐stämme) und deren starker Vermehrung bei oft eingeschränkter Immunantwort zu ausgedehnter, auch ischämisch bedingter Zerstörung des Gewebes kommt, von der Epidermis bis zur tiefen Muskulatur^21^, ^22^ − Toxin‐bedingte Schocksymptomatik und unmittelbare vitale Bedrohung − Leit‐ und einziges typisches Frühsymptom: Überproportional ausgeprägte Schmerzen, das heißt stärker als der klinische Befund vermuten lässt, auf Ischämie und Nekrosen zurückgehend; crescendoartig, nur auf Morphine ansprechend
***Gangrän***
− Nekrosen an Zehen, Ödem, kalte Haut bei Erythem des distalen Fußes, bei deutlichem Ödem und Flüssigkeit in der Haut des Zehenzwischenraumes auch feuchte Gangrän − septische Gangrän = Verschluss der versorgenden Arterie infolge eines Infektes (klinisches Bild des bläulichen „*sausage toe*“) und ischämische, typischerweise trockene Gangrän, die sich sekundär infizieren kann

Die hier genannten Differenzialdiagnosen können Folge oder Komplikationeiner infizierten Zehenzwischenraum‐Intertrigo werden. Insbesondere Fußinfektionen bei Patienten mit insuffizient eingestelltem Diabetes mellitus, sei es als DFU oder Zehenzwischenraum‐Intertrigo, werden leicht zur komplizierten Phlegmone und bedürfen besonderer Aufmerksamkeit, da sie sich schnell in tiefe Gewebeschichten, Knochen (Osteomyelitis) und in den Blutstrom ausbreiten können.

## DIAGNOSTIK

### Initiale klinische Diagnosestellung

**Table:** 

**Empfehlung**
Die initiale Diagnose lässt sich **klinisch** anhand folgender Untersuchungsbefunde stellen:
− flächige Erosionen im Verlauf gegebenenfalls auch Ulzerationen vom Zehenzwischenraum gegebenenfalls bis zum Fußrücken − gelbes Exsudat, gelb‐grünliche Beläge, gegebenenfalls Eiter − Mazeration am Rand des Barrieredefekts − süßlich‐fauliger Geruch − Ödem − Schmerzen − fehlende Heilungstendenz, oft allmähliche Größenzunahme (Eiter oder fehlende Heilung, Größenzunahme und Schmerzen und zunehmende Rötung des Wundrandes sind die Kriterien für eine lokale Infektion von Ulzera) − oft mazerierende interdigitale Fußmykose oder andere vorangegangene Traumata − typische Begleitkrankheiten (Komorbidität) in der Anamnese, welche die lokale Infektion begünstigen, wie chronisches Lymphödem, Diabetes mellitus, Polyneuropathie oder pAVK

### Erregerdiagnostik

**Table:** 

**Empfehlung**
Zur Bestätigung der klinischen Diagnose **kann** ein **Erregernachweis inklusive Resistenztestung** erfolgen. Bei komplizierten beziehungsweise nekrotisierenden Prozessen **soll** ein **Erregernachweis inklusive Resistenztestung** erfolgen.
− Bei der Interpretation eines oberflächlichen Wundabstrichs ist zu beachten, dass dieser wenig aussagekräftig ist, da auch kolonisierende und sekundär infizierende Bakterien erfasst werden. − Wenn ein Wundabstrich erfolgt, sollte dieser möglichst an infizierten Wundrändern, am Wundgrund oder am Gewebe von Wundkrusten erfolgen. − Der mikrobiologische Nachweis gramnegativer Stäbchen (am häufigsten *Pseudomonas aeruginosa* und *Enterobacterales*) zusätzlich zum Nachweis von *S. aureus* oder β‐hämolysierenden Streptokokken ist zwar bei der infizierte Zehenzwischenraum‐Intertrigo typisch, hat aber sonst keine weitere klinische Relevanz. − Wenn indiziert und gewinnbar, vor allem jedoch bei Zeichen einer komplizierten (schweren) Phlegmone und nekrotisierenden Infektionen sollen native Gewebeproben (Biopsien) aus Wundrändern oder Wundgrund vor Abstrichen bevorzugt werden, da aus ihnen am sensitivsten und spezifischsten die Erreger der Weichgewebeinfektion kulturell oder DNA‐basiert nachgewiesen werden können; eine Anzucht gelingt am besten vor Antibiotikagabe.

Die für die Infektion ursächlichen und vitalen Erreger befinden sich an den Wundrändern, am Wundgrund beziehungsweise unter Wundkrusten. Ein Abstrich sollte möglichst von verschiedenen Lokalisationen erfolgen (ohne Berührung von nicht‐lädierten Bereichen des Wundrandes). Oberflächliche Abstriche aus Erosionen beziehungsweise Ulzera sind somit wenig aussagekräftig und führen wegen Fehlinterpretation zu unsachgemäßer antimikrobieller Therapie.^23^ Wird dennoch ein Abstrich durchgeführt, sollten sogenannte „geflockte“ Nylontupfer mit Amies‐Transportmedium genutzt werden. Oberflächliche Sekrete und fibrinöse beziehungsweise nekrotische Beläge sind vorher steril zu entfernen.

Wenn indiziert und gewinnbar, sind sensitivitätsbedingt, *native* Gewebeproben zu bevorzugen, da sie neben der Kultivierung der vermeintlich relevanten Bakterien auch den Einsatz nukleinsäurebasierter Nachweisverfahren ermöglichen, entweder parallel oder nachträglich bei negativer Kultur. Die Gewinnung von Gewebeproben ist aber eher dann sinnvoll, wenn es klinische Zeichen für eine Ausbreitung der Infektion in das Weichgewebe (Dermis, Subkutis oder tiefere Schichten) gibt (also bei Phlegmone), und wenn noch keine Antibiotikagabe erfolgt ist (da sonst erfahrungsgemäß die Anzucht auch von in vitro resistenten Bakterien nicht gelingt). Sie ist am ehesten indiziert, wenn eine schwere oder sich schnell ausbreitende komplizierte Phlegmone vorliegt. Hierzu wird empfohlen, die Gewebeproben zu teilen und Teilproben zwischenzulagern (je nach Zeitdauer 5 °C bis ≤ –20 °C). Werden parallel histopathologische Untersuchungen notwendig, ist das Gewebematerial für die Pathologie und Mikrobiologie (nicht formalinfixiert) aufzuteilen. Stehen nur formalinfixierte/paraffinierte Gewebeproben zur Verfügung, können bei deutlich gesenkter Sensitivität und erhöhter Kontaminationsgefahr molekulare Verfahren im Sinne eines „*Rescue*‐Verfahrens“ eingesetzt werden.^24^ Bei Ulzerationen sind steril entnommene Exzisionsproben am besten geeignet.^23^ Die Gewebeproben sollten aus dem geröteten, infizierten Areal entnommen werden, etwa 1 cm vom Wundrand entfernt, nach vorheriger gründlicher antiseptischer Reinigung der Hautoberfläche, um eine Kontamination der Probe mit Erregern aus dem Ulkus zu vermeiden. Es soll ermittelt werden, welche Bakterien unterhalb der intakten Epidermis für die Weichgewebeinfektion verantwortlich sind.^23^ Angesichts der inhomogenen Verteilung von Erregern im infizierten Gewebe ist nahe dem Wundrand eine verhältnismäßig höhere Erregerdichte zu erwarten.

Bei Gewebeproben müssen sterile Transportbehälter eingesetzt werden. Eine Austrocknung der Gewebeproben muss durch Zusatz steriler physiologischer Kochsalzlösung verhindert werden. Transport‐ und Lagerungszeiten mindern die diagnostische Ausbeute. Transportzeiten sind deshalb kurz zu halten, optimal unter zwei Stunden. Bei Überschreiten von 2–4 Stunden Transportzeit sollte der Probentransport in der Regel gekühlt erfolgen.

Die kulturelle Anlage sollte eine breite Erregerpalette berücksichtigen einschließlich Anaerobier (siehe Mikrobiologisch‐infektiologische Qualitätsstandards (MiQ) „Infektionen der Haut und der subkutanen Weichgewebe“,^23^). Die beimpften Agarplatten werden 48 Stunden bebrütet, eine erste Ablesung erfolgt nach 24 Stunden. Die Bouillonröhrchen werden über 7 Tage täglich auf Wachstum kontrolliert.

Zum Nachweis seltener Erreger sowie auf diagnostischen Strategien zum Screening beziehungsweise Nachweis von multiresistenten Erregern (MRE) sei auf die Literatur verwiesen.^23^

Bei der Interpretation der mikrobiologischen Befunde ist es wichtig, zu beachten, dass *(1)* es grundsätzlich bei Probengewinnung aus primär nichtsterilen Bereichen schwierig ist, zwischen Kolonisation und Infektion, bei nicht sachgerechter Probengewinnung auch Kontamination, zu unterscheiden, *(2)* die Erregerverteilung im infizierten Gewebe inhomogen ist und *(3)* die Vitalität und damit Anzüchtbarkeit der Bakterien je nach Stärke der Immunantwort oder nach einer vorherigen Antibiotikabehandlung (selbst bei möglicher Resistenz) herabgesetzt ist.

### Einschätzung des Schweregrades

Für die Beurteilung beziehungsweise Graduierung des Schweregrades einer infizierten Zehenzwischenraum‐Intertrigo liegen derzeit keine validierten Stadieneinteilungen vor.

Lokalisation, Ausprägung und Ausdehnung der Mazerationen, des Erythems, der Ödeme und Erosionen sowie Ulzerationen sollten beschrieben werden. Eine Fotodokumentation erleichtert die Verlaufsbeurteilung.^25^ Zur besseren Vergleichbarkeit und Vorhersagbarkeit sollte eine strukturierte Beschreibung der Wundverhältnisse erfolgen. Hilfreich ist hierzu die Verwendung einer der gängigen Wundklassifikationen.

Unterschieden werden sollte die lokale Infektion in Form der infizierten Zehenzwischenraum‐Intertrigo von der – mitunter davon ausgehenden – Weichgewebeinfektion in Form der Phlegmone.

Zur Beschreibung des Ausmaßes (zum Beispiel im Rahmen von Studien) ist die international anerkannte PEDIS‐Klassifikation der *International Working Group on the Diabetic Foot* (IWGDF) geeignet,^26^ die primär aber für Fußinfektionen bei Diabetes mellitus gilt und entsprechend dort relevante Parameter beschreibt: Perfusion, flächenmäßige Ausdehnung und Tiefe der Wunde, vorliegende lokale Entzündungszeichen und Empfindungsstörung. Sie wird auch in der deutschsprachigen Leitlinie zur Behandlung von Haut‐ und Weichgewebeinfektionen genutzt.^2^

In der jüngsten Aktualisierung favorisiert die IWDGF die Verwendung des SINBAD Klassifizierungssystem und ‐score. Es handelt sich um ein Akronym, das aus sechs Elementen besteht, die nach ihrem Schweregrad abgestuft sind: Ulkusstelle, Ischämie, Neuropathie, bakterielle Infektion, Fläche und Tiefe. Der Gesamtwert liegt zwischen 0 und 6 und ist in drei Kategorien unterteilt, die sich auf das Risiko einer Amputation der unteren Gliedmaßen beziehen.^27^ Falls die klinische (ischämische Ruheschmerzen, kühle Haut, verlängerte Rekapillarisierungszeit) und apparative Diagnostik einen Hinweis auf das Vorliegen einer pAVK erbracht hat, so kann der Benefit einer vaskulären Intervention und das Risiko einer gegebenenfalls drohenden Amputation mit dem WIFI‐System abgeschätzt werden, welches die Kriterien Wunde, Ischämie und Fußinfektion beinhaltet (wound, ischemia, foot infection; WIFI). Es basiert auf der Klassifikation der *Infectious Disease Society of America* (IDSA)/*International Working Group on the Diabetic Foot* (IWGDF) zur Erfassung der Infektion. Amputationsraten und die Abheilungsdauer der Wunde korrelieren mit WIFI. Das WIFI‐System wird vor allem wissenschaftlich genutzt. Zur Beurteilung von Patienten mit Diabetes fehlt in diesem System jedoch das Kriterium „Neuropathie“.^28^, ^29^

Zur Beurteilung der Fußinfektion bei Menschen mit Diabetes mellitus empfiehlt die IWGDF von den beiden Systemen aktuell:

− die Klassifikation SINBAD für den klinischen Gebrauch und für den Vergleich von Ergebnissen (Audit),
− das WIFI‐System zur Erfassung von Infektion und Perfusion und zur Abschätzung des Vorteils einer Revaskularisation.^27^, ^30^, ^31^

Die schwerste Form der Weichgewebeinfektionen stellen die sogenannten nekrotisierenden Weichgewebeinfektionen mit unmittelbar vitaler Bedrohung dar, die Verdachtsdiagnose wird klinisch gestellt (siehe Kapitel „Differenzialdiagnosen“). Eine Hilfe zur Unterscheidung von komplizierten, nichtnekrotisierenden Phlegmonen kann der *Laboratory Risk Indicator for Necrotizing Fasciitis (LRINEC) Score* bieten.^32^, ^33^, ^34^, ^35^

Kein Klassifikationssystem kann die Prognose und den Verlauf der Infektion oder eines Ulkus sicher vorhersagen.

### Erweiterte diagnostische Maßnahmen

**Table:** 

**Empfehlung – Ausbreitungsdiagnostik**
− Regelmäßige klinische Kontrolle, um Hinweise auf eine Weichgewebeinfektion (meist Phlegmone) rechtzeitig zu erkennen zum Beispiel sich ausbreitendes Erythem oder Schwellung. − Neben dem klinischen Befund ermöglichen CRP und Leukozyten einen Hinweis auf eine Phlegmone und eine Bewertung des Therapieansprechens (unter Berücksichtigung der typischen Latenz, bei CRP 48–72 Stunden). − Bei Fußinfektionen der Patienten mit insuffizient eingestelltem Diabetes mellitus können sich Phlegmone schnell in die Tiefe ausbreiten, so dass entsprechende klinische und bildgebende Ausbreitungsdiagnostik früh erfolgen sollte.^2^, ^36^, ^37^ − Bei Verdacht auf tiefer reichende Infektion mit Beteiligung von Sehnen, Gelenkkapsel oder Knochen oder bei starken Schmerzen weitere Ausbreitungsdiagnostik mit bildgebenden Verfahren. − Vor Beginn einer Antibiotikatherapie soll nach Verdachtsmomenten für eine Osteomyelitis geschaut werden.^38^ − Verdachtsmomente für eine Osteomyelitis sind:^38,^ ^39^ ○ Ulzera, die trotz angemessener Wundbehandlung und bei Fehlen einer relevanten Ischämie oder pAVK nicht abheilen ○ Ulzera mit einer Ausdehnung von mehr als 2 cm^2^ und 3 mm Tiefe^38^ ○ Knochenkontakt bei der Sondierung, ○ frei liegende Knochenanteile ○ bakterielle Daktylitis (wurstförmig geschwollene Zehen) ○ pathologische BSG (> 70 mm), erhöhtes CRP, erhöhte Procalcitonin‐Werte, Leukozytose

**Table:** 

**Empfehlung ‐ Bildgebung**
Bei entsprechenden Symptomen für eine klinisch vermuteter Ausdehnung der Infektion **soll** eine Bildgebung des betroffenen Fußes veranlasst werden.
− Bei umschriebener Abszedierung kann eine Sonographie ausreichend sein. − Bei tiefreichender Infektion unklarer Ausdehnung und dringendem Verdacht auf das Vorliegen einer Osteomyelitis ist eine Schnittbilddiagnostik geeignet, um die Gesamtausdehnung eines abszedierenden oder phlegmonösen Geschehens mit möglicher Osteomyelitis zu diagnostizieren. − Ein MRT liefert die beste Aussagekraft. Bei Kontraindikationen für eine MRT‐Untersuchung oder fehlender Verfügbarkeit ist ein CT eine alternative Option.^40^ Wenn eine MRT‐ oder CT‐Untersuchung nicht möglich oder verfügbar sind, sollte zumindest kurzfristig eine Röntgenaufnahme des Fußskeletts in zwei Ebenen zum Ausschluss einer Osteomyelitis erfolgen. − Bei anhaltendem Verdacht auf Osteomyelitis und wenn keine Klärung durch Schnittbildgebung gelungen ist, kann ein ^18^F‐FDG‐PET beziehungsweise eine ^99^mTc‐HMPAO‐markierte Leukozytenszintigraphie erfolgen. − Wenn ein Verdacht auf Osteomyelitis besteht, sollte zur Absicherung eine Knochenbiopsie zur mikrobiologischen und histologischen Untersuchung erfolgen (wenn vorher ein Antibiotikum gegeben wurde erst 15 Tage nach Absetzen der Antibiotikagabe).^38^

**Table:** 

**Empfehlung – Vaskuläre Diagnostik**
Bei der klinischen Untersuchung **soll** eine Abklärung der arteriellen und venösen Versorgung der betroffenen Extremität erfolgen (siehe unten) und bei entsprechendem Verdacht eine Bildgebung einbeziehen. Bei erniedrigtem Knöchel‐Arm‐Druckindex, anderweitigem klinischen Verdacht auf Gefäßpathologien oder ausbleibender Befundbesserung unter adäquater Therapie ohne erkennbaren Grund **soll** eine angiologische oder gefäßchirurgische Vorstellung erfolgen. Es wird auf die S3‐Leitlinie „Periphere arterielle Verschlusskrankheit (pAVK), Diagnostik, Therapie und Nachsorge“ (AWMF‐Registernummer: 065‐003) sowie das gemeinsame Positionspapier der diabetologischen, angiologischen, interventionell‐radiologischen und gefäßchirurgischen Fachgesellschaften verwiesen.^41^ Einzelne Maßnahmen werden hier aufgelistet:
**Arteriell**
− Um das Vorliegen einer pAVK abzuklären, soll der Knöchel‐Arm‐Druckindex (Ankle‐Brachial‐Index; ABI) bestimmt werden (systolische Dopplerverschlussdruckmessung in Ruhe an beiden Oberarmen und distalen Unterschenkeln, normal 0,9–1,3). − Bei ABI‐Werten > 1,3 mit Verdacht auf eine Mediasklerose (bei circa 30% der Menschen mit Typ‐2‐Diabetes), falsch erhöhter Wert aufgrund der eingeschränkten Kompressibilität), anderweitig nicht plausiblen Verschlussdrücken oder anderen Hinweisen auf eine pAVK (kalte Zehen, Irisblendenphänomen, anamnestische Hinweise) sollen ergänzende Methoden wie Zehenverschlussdruckmessung (TBI), Analyse des pedalen Dopplerfrequenzspektrums oder eine Oszillographie eingesetzt werden. − Ab ABI < 0,9, TBI < 0,7 oder monophasischem Dopplerfrequenzspektrum sollte eine weiterführende angiologische Diagnostik durchgeführt werden. − Im Falle einer kritischen Ischämie (ABI < 0,5, transkutane Sauerstoffpartialdruckmessung tcpO_2_ < 30 mmHg) besteht akuter Handlungsbedarf zur Vermeidung ischämischer Komplikationen. − In der weiterführenden Diagnostik nimmt die farbkodierte Duplexsonographie (FKDS) der Bein‐ und Beckenarterien eine Schlüsselrolle zur Prüfung der Notwendigkeit und Planung revaskularisierender Maßnahmen ein. Anhand ihrer Ergebnisse soll über die Indikation zur Durchführung einer Angiographie in Interventionsbereitschaft oder zusätzlich bildgebender Verfahren wie MR‐ oder CT‐Angiographie entschieden werden.
**Venös**
− FKDS der Becken‐ und Beinvenen ist bei Verdacht auf Phlebothrombose indiziert.

### Abklärung von Begleiterkrankungen

**Table:** 

**Empfehlung**
Bei der infizierten Zehenzwischenraum‐Intertrigo **sollte** auf klinische Hinweise für das Vorliegen folgender Erkrankungen geachtet und gegebenenfalls weitere diagnostische und therapeutische Schritte veranlasst werden:
− Diabetes mellitus − Lymphödem − chronische venöse Insuffizienz − pAVK

Untersuchungen zeigen, dass Fußinfektionen gehäuft bei Menschen mit Diabetes mellitus auftreten. So wurde zum Beispiel in dem mit 200 Betroffenen größten israelischen Studienkollektiv zu infizierten Zehenzwischenraum‐Intertrigo ein Anteil von 16% von Betroffenen mit Diabetes mellitus beschrieben. Auffällig war, dass diese im Vergleich zu Menschen ohne Diabetes mellitus signifikant höhere CRP‐Werte aufzeigten. Dies weist daraufhin, dass Menschen mit Diabetes dazu neigen, schwerere Krankheitsverläufe zu entwickeln. Andere Begleitkrankheiten wie arterielle Hypertonie oder Dyslipidämie scheinen nicht die Schwere der Erkrankung zu beeinflussen.^6^ Menschen mit Diabetes mellitus sind aufgrund von Hyperglykämie‐induzierten metabolischen und immunologischen Veränderungen wie zum Beispiel einer verminderten T‐Zell‐Reaktion und Neutrophilenfunktion und aufgrund der pAVK beziehungsweise okkludierenden Arteriolopathie anfälliger für Hautinfektionen. Das hyperglykämische Wundmilieu fördert die Pathogenität von *S. aureus*.^42^ Die oft vorliegende diabetische Neuropathie führt zu einer geringeren Schmerzwahrnehmung und hierdurch zu einem unbemerkten Fortschreiten einer Infektion, die sich zudem schnell (über Sehnen) von Kompartimenten mit hohem in Kompartimente mit niedrigeren Gewebedrücken ausbreitet (siehe oben). Die klinische Befunderhebung sollte eine Untersuchung auf eine mögliche pathologische Druckbelastung der Fußhaut durch eine neuropathisch bedingte Fußdeformität und eine Sensibilitätsuntersuchung beziehungsweise Prüfung auf Vorliegen einer sensorischen Neuropathie mittels Stimmgabel, TipTherm^®^ und Monofilament enthalten. Das Risiko für schwere Infektionen ist bei vorangegangenen Fußläsionen oder Amputationen erhöht.

## THERAPIE

### Lokale Therapie

**Table:** 

**Empfehlung**
Die Lokaltherapie **soll** unabhängig vom Schweregrad der Zehenzwischenraum‐Intertrigo durchgeführt werden. Es **sollten** die folgenden übergeordneten Therapieziele verfolgt werden:
Reduktion der Inflammation Reduktion der Bakterien und gegebenenfalls Pilze Reduktion der „feuchten Kammern“ Reduktion des Ödems

**Ad 1)** Zu Beginn der Therapie werden lokale Therapien mit Umschlägen beispielsweise mit synthetischen Gerbstoffen wie dem Tannin Tamol empfohlen, in Kombination mit Antiseptika (siehe Ad 2). Diese Gerbstoffe wirken antinflammatorisch und adstringierend.^43^ Kurzzeitig ist bei starker entzündlicher Aktivität auch der topische Einsatz hochpotenter Glukokortikoide sinnvoll.^44^ Bei der Auswahl der genutzten Galenik ist der Grundsatz „feucht auf feucht, trocken auf trocken“ zu beachten (Ausnahmen siehe Ad 3.). Somit kommen in der Akutbehandlung in erster Linie alkoholfreie Lotionen zum Einsatz.

**Ad 2)** Durch ein mechanisches Débridement, beispielsweise mit sterilen Baumwollkompressen, kann bereits ein Teil der Bakterien und Wundbeläge effektiv entfernt werden.^45^, ^46^ Eine Alternative stellen spezielle Reinigungspads oder Schwämme dar.^47^ Um die Anzahl der Bakterien und Pilze weiter zu verringern, werden moderne, wenig zytotoxische Antiseptika mit Polihexanid (PHMB) oder Octenidin (mit Phenoxyethanol), gegebenenfalls auch Povidon (PVP)‐Jod eingesetzt, die beispielsweise mit feuchten Umschlägen appliziert werden.^48^ Hier sind die jeweiligen Einwirkzeiten der Antiseptika (PHMB 10–20 Minuten, Octenidin 1–2 Minuten, PVP‐Jod 3–5 Minuten.) zu beachten.^49^ Der Zusatz von Wirkstoffen wie Kaliumpermanganat, Farbstoffen, Wasserstoffperoxid oder Chinolinol wird von der Leitliniengruppe nicht empfohlen, weil sie zelltoxischer sind, beim Ansetzen beziehungsweise Verdünnen leicht Fehler passieren können und weil die Arzneistoffqualität und ‐quantität der Farbstoffe nicht sichergestellt werden kann. Auch topische Antibiotika wie beispielsweise Gentamycin sollten ebenso wie Silbersulfadiazin nicht mehr eingesetzt werden, da sie unzureichend auf Antibiotika‐resistente Bakterien wirken, möglicherweise resistente kolonisierende Bakterien selektionieren und eine Immunaktivierung mit nachfolgender Arzneimittelreaktion vermitteln können.^48^ Wenn eine Tinea pedis nachgewiesen wurde, sollte eine topische antimykotische Behandlung spätestens nach Beendigung der antiseptischen Therapie erfolgen.

***Ad 3)*** Im Gegensatz zu vielen anderen Wunden werden bei Infektionen der oft feuchten Zehenzwischenräume (vor allem wenn der Einfluss gramnegativer Bakterien anhand von Geruch und grünlichem Wundexsudat und Eiter klinisch erkennbar ist) meist austrocknende Behandlungsmaßnahmen durchgeführt, um das für Feuchtkeime begünstigende lokale Milieu zu begrenzen. Hier hat die Einlage von Baumwollkompressen oder anderen weichen Textilstoffen in die Zehenzwischenräume eine wichtige Bedeutung, um die Ausbildung sogenannter feuchter Kammern beziehungsweise von Intertrigo zu verhindern und Feuchtigkeit nach außen zu transportieren.^50^ Hierfür gibt es auch medizinische Textilstoffe, die antimikrobiell wirksames Silber beinhalten.^51^ Bei sehr exsudativen Wunden sollten noch Exsudat‐bindende Sekundärverbände bis hin zu Superabsorbern genutzt werden.^52^ Das Gewebe sollte aber nicht zu trocken werden, da ein feuchtes Wundmilieu für die meisten an der Wundheilung beteiligten Prozesse notwendig ist.

***Ad 4)*** Bei ausgeprägten Ödemen sollte, nach Ausschluss von Kontraindikationen wie fortgeschrittener peripherer arterieller Verschlusskrankheit (ABI < 0,5; Knöchelarteriendruck < 60 mmHg, Zehendruck < 30 mmHg, TcPO_2_ < 20 mmHg^53^), dekompensierter Herzinsuffizienz (NYHA III + IV) und Phlegmasia coerulea dolens, eine medizinische Kompressionstherapie durchgeführt werden.^54^ Meist ist für die effektive Ödemreduktion ein geringer Anlagedruck um 20 mmHg bei einem Beginn der Kompression an den Grundgelenken der Zehen bis zu den Knien ausreichend.^55^

Beispielhafte Darstellung des praktischen Ablaufs der Lokaltherapie eines akuten, inflammatorischen gramnegativen Fußinfekts:

1. Feuchte Umschläge über circa 10 Minuten mit Zusatz, zum Beispiel von Gerbstoffen
2. Mechanische Säuberung zum Beispiel mit sterilen Baumwollkompressen
3. Anlage feuchter Umschläge, zum Beispiel mit Octenidin und Phenoxyethanol für mindestens zwei Minuten
4. dünnes Auftragen zum Beispiel einer Betamethason‐Lotion
5. Einbringen steriler Baumwollkompressen in die Zehenzwischenräume, gegebenenfalls mit Sekundärverband
6. Bei ausgeprägtem Ödem Anlage eines medizinischen Kompressionsverbands mit mindestens 20 mmHg Anlagedruck

Die Verbandwechselintervalle orientieren sich an den jeweiligen Exsudatmengen. Zu Beginn der Therapie kann ein Wechsel mehrfach am Tag notwendig sein. Im weiteren Verlauf der Erkrankung sind die Punkte 1, 4 und 6 oft entbehrlich; die Verbandwechsel müssen dann nicht mehr täglich erfolgen.

Bei begleitender plantarer Hyperhidrose kann längerfristig zudem eine Iontophorese erwogen werden.^56^

### Systemische Therapie

**Table:** 

**Empfehlung**
Eine systemische antibiotische Therapie **soll** bei **zusätzlichem Vorliegen einer Weichgewebeinfektion** (meist unkomplizierte oder komplizierte Phlegmone [siehe Kapitel Differenzialdiagnosen]) entsprechend der S2k‐Leitlinie „Kalkulierte parenterale Initialtherapie bakterieller Erkrankungen bei Erwachsenen – AWMF‐Registernummer 082 – 006“* durchgeführt werden. Daher soll die antibiotische Therapie vor allem gegen *S. aureus* wirksam sein (Cefazolin oder Flucloxacillin parenteral). Als Therapiedauer werden allgemein 5 Tage empfohlen.^39,^ ^57^, ^58^ Die Therapiedauer soll sich aber auch nach dem klinischen Ansprechen richten. *Die S2k Leitlinie wurde 2017 verfasst und befindet sich aktuell in Überarbeitung. Eine systemische antibiotische Therapie ist bei der infizierten Zehenzwischenraum‐Intertrigo nicht generell indiziert, da es sich primär um eine oberflächlich infizierte Erosion oder Ulzeration handelt. Ein kultureller Erregernachweis aus Wundabstrichen rechtfertigt daher ohne klinische Zeichen einer Weichgewebeinfektion keine systemische antibiotische Therapie. Wenn eine Phlegmone schnell progredient sein sollte oder auf obige Therapie nicht anspricht, wird wegen möglicher gramnegativer Bakterien in der infizierten Intertrigo (und des oft vorliegenden Diabetes mellitus) ein Aminopenicillin plus Beta‐Lactamase‐Inhibitor (Amoxicillin/Clavulansäure oder Ampicillin/Sulbactam) empfohlen.
Im Verlauf *sollte* die antibiotische Therapie nach Vorliegen eines Resistogramms aus Gewebeproben (Siehe Kapitel „Erregerdiagnostik“) reevaluiert und eventuell angepasst werden.

In der Vergangenheit wurden zur systemischen Therapie der gramnegativen Fußinfektion beziehungsweise Zehenzwischenraum‐Intertrigo regelhaft Fluorchinolone angewandt. Diese werden aber wegen der in der Regel fehlenden Indikation sowie geringer therapeutischer Breite^2^ nicht mehr empfohlen. Es besteht zudem ein erhöhtes Risiko für beeinträchtigende und irreversible muskuloskelettale und neurologische Nebenwirkungen (Durchführungsbeschluss der EU‐Kommission C(2019)2050 und Risikobewertungsverfahren nach Art. 31 der Richtlinie 2001/83/EG).

Die Bioverfügbarkeit oraler Antibiotika ist aus den oben genannten Gründen nicht ausreichend sicher. Eine Lokaltherapie mit Antibiotika ist abzulehnen, weil sie nicht sicher genug wirksam ist, resistente Bakterien herausselektioniert und über das Immunsystem der Haut eine Sensibilisierung gegenüber der entsprechenden Antibiotikagruppe verursachen kann. Bei den genannten Antiseptika hingegen ist die topische Wirksamkeit besser gesichert und eine Resistenzentwicklung weniger wahrscheinlich.

Eine Literaturrecherche zu Weichgewebeinfektionen bei infizierter Zehenzwischenraum‐Intertrigo wurde durchgeführt, war wegen der engen Fragestellung, des oft unscharfen Gebrauchs der Begriffe (lokale Infektionen, komplizierten oder unkomplizierten Phlegmone) und auch wegen der unterschiedlichen mikrobiologischen Untersuchungen (Abstriche versus Gewebeproben) aber nicht ergiebig und entsprechende Ergebnisse sind nicht immer gut vergleichbar.^4^ Beispielhaft seien hier Auszüge einer orientierenden Literaturrecherche genannt, die im Rahmen der Leitlinienarbeit durchgeführt wurde. Die Heterogenität der Versorgung und der unseres Erachtens nicht notwendige Einsatz von Antibiotika mit breitem Spektrum und hohem Risiko unerwünschter Arzneimittelwirkungen werden deutlich:

In einer retrospektiven multizentrischen französischen Studie zu *Gram­negative Toe Web Infection* erhielten 45,2% der Patienten (gesamt n = 62) zusätzlich zur Lokaltherapie eine systemische antibiotische Behandlung. Bei 57% richtete sie sich gegen *Pseudomonas aeruginosa* und führte zur Gabe von Piperacillin‐Tazobactam mit oder ohne Ciprofloxacin.^44^ Ein anderer Teil erhielt hingegen Antibiotika gezielt gegen *S. aureus* oder β‐hämolytische Streptokokken, einschließlich der Patienten mit “Cellulitis” (nicht näher definiert) (24%) mit nicht geringerem Behandlungserfolg.^44^ In einer prospektiven monozentrischen Studie wurden 123 Patienten systemisch mit Ciprofloxacin oder intramuskulärer Injektion von Ceftazidim beziehungsweise Cefotaxim behandelt, aber die Anzahl der erfolgreich behandelten Patienten nicht nach den einzelnen Therapeutika stratifiziert.^5^

Die Leitliniengruppe verweist für die Auswahl antibiotischer Systemtherapien auf die S2k‐Leitlinie *„Kalkulierte parenterale Initialtherapie bakterieller Erkrankungen bei Erwachsenen“ AWMF‐Registernummer 082‐006*.*59* Dort insbesondere auf das Kapitel 9 ab Seite 173 zu Haut‐ und Weichgewebeinfektionen (eine Aktualisierung ist in Arbeit).^2^ Entsprechend wäre in vielen Fällen zunächst eine antibiotische Therapie gegen *S. aureus* angezeigt (ab Seite 183 berichtet).^2^ ^,39^ Dieses Vorgehen hat sich im klinischen Alltag der Autoren der vorliegenden Leitlinie bewährt.

### Chirurgische Optionen

**Table:** 

**Empfehlung**
Die multimodale Lokaltherapie (siehe Kapitel „Lokale Therapie“) und im Falle einer Weichgewebeinfektion die Antibiotikagabe (siehe Kapitel „Systemische Therapie“) **soll** bei Behandlung der Zehenzwischenraum‐Intertrigo im Vordergrund stehen. Bei den folgenden Begleitumständen oder Verdachtsdiagnosen **soll** eine chirurgische Evaluation erfolgen:
− tiefer reichende Infektion (Befall tiefergelegener Weichgewebe wie zum Beispiel Faszien oder Muskelschichten, analog der Definition komplizierte Haut‐ und Weichgewebsinfektionen^2^, ^60^, ^61^ − Osteomyelitis − schwere Durchblutungsstörungen der Extremitäten − klinische Anzeichen eines Kompartmentsyndroms − Zeichen einer nekrotisierenden Weichgewebeinfektion (siehe Kapitel „Differenzialdiagnosen“)

Die chirurgische Therapie umfasst ein chirurgisches Débridement entzündeter und gegebenenfalls bereits nekrotischer Haut oder gegebenenfalls auch die Amputation von Zehen oder des ganzen Zehenstrahls bei ischämischer Gangrän oder bei tiefer reichender und systemischer Infektion.^22^, ^62^

Wegen der Anatomie des Fußes mit den langstreckigen Muskel‐ und Sehnenkompartimenten können sich Infektionen leicht nach proximal ausbreiten. Die Entzündungsreaktion – verursacht durch die Infektion – kann zu einer Steigerung des Kompartmentdrucks führen, die eine bereits vorliegende Gewebeischämie verschlimmern oder gegebenenfalls diese auch erst hervorrufen kann. Ein klassisches Kompartmentsyndrom tritt zwar eher bei jungen Männern (< 30 Jahren) nach Frakturen oder Traumata auf, aber Varianten wurden auch bei bestimmten ungünstigen Konstellationen oder Kofaktoren (Alkoholabusus, Infektion) beobachtet.^63^ Klassische Symptome sind: schnell verstärkende, unverhältnismäßig heftige Schmerzen einer Gliedmaße, die nicht zu dem äußerlichen Befund passen, derb bis fest tastbarer Muskel, Dehnungsschmerz der betreffenden Muskelloge, sensorische und motorische Nervenausfälle. Differenzialdiagnostisch müssen bei plötzlichen schweren, durch NSAID (*non‐steroidal anti‐inflammatory drugs*) nicht beherrschbaren Schmerzen auch eine nekrotisierende Weichgewebeinfektion oder eine arterielle Embolie (dann auch Blässe des Gewebes, Pulslosigkeit und Parästhesien) ausgeschlossen werden.

Die nekrotisierende Weichgewebeinfektion (NSTI) ist selten, aber Diabetes mellitus beziehungsweise diabetische Ulzera am Fuß sind hierfür ein Risikofaktor,^22^, ^64^, ^65^ neben Dekubitalulzera, höherem Alter und Immunsuppression. Leit‐ und einziges typisches Frühsymptom ist auch hier ein überproportional ausgeprägter, crescendoartiger, nur auf Morphine ansprechender Schmerz, der vor allem durch die Ischämie verursacht wird.^2^ Aufgrund dieser Gefahr ist eine zeitnahe chirurgische Beurteilung einer Weichteilinfektion vor allem bei durchblutungsgestörter Extremität unabdingbar.^22^ Zur Abschätzung des Ausmaßes der Weichteilinfektion sollte eine standardisierte Beurteilung (zum Beispiel WIFI‐Score) erfolgen (siehe auch Kapitel „Einschätzung des Schweregrades“).^22^, ^25^, ^28^, ^29^, ^60^, ^62^, ^66^, ^67^

### Prophylaxe – Behandlung einer Tinea pedis

**Table:** 

**Empfehlung**
Bei bestehender Tinea pedis und/oder Onychomykose **soll** zur Prophylaxe der Zehenzwischenraum‐Intertrigo eine antimykotische Therapie erfolgen.

Eine Tinea pedis interdigitalis gilt als ein prädisponierender Faktor für Mazerationen und für eine sekundäre Besiedelung mit gramnegativen Bakterien (Schädigung der Hautbarriere, Verdrängung der Mikrobiota durch Produktion antibakterieller Substanzen mit Förderung gramnegativer Bakterienkolonisation).^4^ Überdies berichteten in einer Fallserie alle 15 Patienten mit sogenanntem gramnegativem Fußinfekt anamnestisch von rezidivierenden Tinea‐pedis‐Erkrankungen.^6^ *Trichophyton (T.) rubrum* und *T. interdigitale* sind die häufigsten Erreger, während Hefepilze vereinzelt und Schimmelpilze in der Regel nicht nachgewiesen werden.^23^, ^68^ Die Behandlung erfolgt in erster Linie topisch. Eine Ausnahme bilden die Tinea pedis vom dyshidrosiformen Typ und die „Mokkasin“‐Form, die zusätzlich oral mit Terbinafin über 14 Tage behandelt werden.^69^, ^70^ Prinzipiell können alle zur Verfügung stehenden Substanzklassen topischer Antimykotika verwendet werden. Das Allylamin Terbinafin besitzt den Vorteil einer im Regelfall nur einmaligen täglichen Anwendung mit kurzer Therapiedauer über 7 Tage. Während Terbinafin keine zusätzliche antibiotische Wirkung gegenüber gramnegativen Erregern aufweist,^71^ ist sie für Ciclopirox belegt,^72^, ^73^, ^74^ und stellt somit eine Therapieoption dar. Die Applikation erfolgt morgens und abends bis 1–2 Wochen nach Abklingen der Entzündung, in der Regel 3–4 Wochen.^75^ Schuhe und Strümpfe oder Socken sollten initial entsprechend mit antimykotischem Spray und antimykotischem Waschmittel mitbehandelt werden. Präventive Behandlungsmaßnahmen umfassen intensives Abtrocknen, insbesondere der Zehenzwischenräume nach Bad oder Dusche, Tragen sauberer, nichtokkludierender Socken und Schuhe, vorzugsweise aus Baumwolle oder anderen natürlichen Fasern, sowie die Behandlung einer assoziierten Hyperhidrosis plantaris.^4^, ^69^, ^76^

Die Behandlung einer bestehenden Onychomykose wird unter präventiven Gesichtspunkten empfohlen. Es wird zusätzlich auf die S1‐Leitlinie Onychomykose (AWMF‐Registernummer: 013‐003, 2022) verwiesen.^77^

*Für die Abschnitte „Abgrenzung zu anderen Leitlinien/Fächern“, „Limitationen der Leitlinie“, „Forschungsbedarf“, „Informationen zu dieser Leitlinie“ (inklusive „Umgang mit Interessenskonflikten“ und „Finanzierung“) sowie „Methodik“ (inklusive „Generierung von Empfehlungen“, „Verabschiedung der Leitlinie“ und „Verwertungsrechte“) siehe Langfassung*.

## DANKSAGUNG

Open access Veröffentlichung ermöglicht und organisiert durch Projekt DEAL.

## INTERESSENKONFLIKT

Eine vollständige Liste der angegebenen Interessenkonflikte ist verfügbar unter: https://register.awmf.org/assets/guidelines/013‐109l_S1_Infizierte‐Zehenzwischenraum‐Intertrigo‐gramnegativer‐Fu%C3%9Finfekt__2025‐09.pdf
